# Gene Signatures and Prognostic Values of m6A Regulators in Hepatocellular Carcinoma

**DOI:** 10.3389/fgene.2020.540186

**Published:** 2020-10-02

**Authors:** Pei Wang, Xiaotong Wang, Lei Zheng, Chunbo Zhuang

**Affiliations:** ^1^Department of Gastroenterology, The First Affiliated Hospital of Zhengzhou University, Zhengzhou, China; ^2^Department of Clinical Laboratory Medicine, The First Affiliated Hospital of Zhengzhou University, Zhengzhou, China

**Keywords:** hepatocellular carcinoma, m6A, METTL16, TCGA, prognosis

## Abstract

N6-methyladenosine (m6A) is the most abundant mRNA modification in mammals and has been implicated in various biological processes. However, its role in hepatocellular carcinoma (HCC) remains largely unknown. In this study, we investigated the alterations of 19 main m6A regulatory genes in HCC and their association with clinicopathological features, including survival. The mutation, copy number variation (CNV) and clinical data of HCC patients were retrieved from The Cancer Genome Atlas (TCGA) database. We found that the m6A regulators had high frequent alterations in HCC. The alterations of m6A regulators were significantly associated with clinicopathological features as well as TP53 alteration. Patients with any mutation of the m6A regulatory genes had worse overall survival (OS) and disease free survival (DFS). Deletion of METTL16 or ALKBH5 predicted poor OS and DFS of HCC patients. Moreover, deletion of METTL16 was an independent risk factor for DFS. Low METT16 expression was association with activation of multiple metabolic pathways in HCC. Finally, by RT-PCR, we confirmed that METTL16 was downregulated in HCC, and that lower METTL16 expression was associated with poor OS. In conclusion, we reported a significant association between alterations of m6A regulators and clinicopathological features, and highlighted the importance of METTL16 among the 19 m6A regulators in HCC pathogenesis. These findings will provide new insights into the role of m6A modification in HCC.

## Introduction

Hepatocellular carcinoma (HCC) is the second leading cause of cancer-related deaths worldwide, resulting in over 500,000 deaths per year (Torre et al., 2015). Despite the recent advances in surgical resection and liver transplantation, the 5-year survival rate of liver cancer patients is still less than 17% (Miller et al., 2016). In most cases, HCC is detected at advanced stage with limited therapeutic options. Only 10–20% of the tumors are considered surgically resectable at the time of diagnosis (El-Serag, 2011). Thus, a better understanding of the molecular mechanisms underlying the initiation and progression of HCC, as well as identification of prognostic biomarkers are still needed.

N6-methyladenosine (m6A) is the most abundant chemical modification in eukaryotic mRNA (Meyer et al., 2012). The m6A modification is a reversible process regulated by the balanced activities of m6A “writer” and “eraser” proteins (writers, erasers). m6A is installed by an RNA methyltransferase complex, the m6A writer, which is consisted of methyltransferase like 3 (METTL3), METTL14, Wilm’s tumor 1-associated protein (WTAP), KIAA1429, RNA binding motif protein 15 (RBM15), and zinc finger CCCH domain-containing protein 13 (ZC3H13) (Liu et al., 2014). Besides, METTL16 is a newly identified RNA methyltransferase independent from the methyltransferase complex. The reversible process is conducted by the m6A erasers, including fat mass and obesity-associated protein (FTO) and alkB homolog 5 (ALKBH5) (Jia et al., 2011; Zheng et al., 2013). The m6A-modified mRNAs are specifically recognized by the m6A readers YTH domain containing proteins YTHDC1-2, YTH-family proteins YTHDF1-3, insulin-like growth factor 2 mRNA-binding proteins IGF2BP1-3, as well as heterogeneous nuclear ribonucleoprotein HNRNPA2B1 and HNRNPC, leading to alterations of mRNA splicing, export, translation and degradation (Wang X. et al., 2014; Xiao et al., 2016; Meyer and Jaffrey, 2017). M6A modification is implicated in various biological processes including circadian rhythm (Hastings, 2013), lipid metabolism (Zhong et al., 2018), embryonic stem cell differentiation (Wang Y. et al., 2014), as well as carcinogenesis (He et al., 2018). Increasing evidence have indicated that dysregulation of m6A pathway is frequently found in malignancies such as breast cancer (Niu et al., 2019), glioblastomas (Zhang et al., 2017), acute myeloid leukemia (Paris et al., 2019), lung cancer (Liu et al., 2020), and HCC (Lan et al., 2019). It is reported that the m6A modification level of total RNA is decreased in HCC, and that METTL14 is the main factor involved in the aberrant m6A modification (Ma et al., 2017). The decreased METTL14 expression promoted tumor metastasis by reducing the m6A modification level and miR-126 expression. Another study demonstrated that the high expression of METTL3 in HCC led to increased tumor growth and m6A modification level, mediating degradation of the tumor suppressor SOCS2 through an m6A reader protein YTHDF2-dependent pathway (Chen et al., 2018). Knockdown of METTL14 significantly suppressed HCC cell proliferation and migration (Chen et al., 2018). These findings underscore the complexity of m6A modification and its regulatory enzymes in HCC. The role of m6A-related factors in carcinogenesis and progression of HCC remains to be elucidated. Hence, in the present study, we analyzed the clinical and sequencing data of HCC cohort from TCGA datasets, and investigated the alteration spectrum and prognostic values of 19 main m6A regulatory genes in patients with HCC.

## Materials and Methods

### Ethics Statement

We retrieved the mutation, CNV, mRNA expression, and clinical data from The Cancer Genome Atlas (TCGA) database by cBioPortal^1^ platform. All the data are publicly available and open-access. We used only anonymous statistical gene expression.

### Data Processing

A total of 373 HCC patients in the TCGA database with CNV, mutation and clinical data were included in this study. The loss and gain levels of copy number was identified using the Genomic Identification of Significant Targets in Cancer algorithm (GISTIC). To evaluate the clinical significance of the CNV and mutation status, the HCC cohort was divided into two subgroups: “with CNV and/or mutation of the 19 m6A regulatory genes” and “without CNV or mutation.” For separate analysis of the association between clinicopathological feature and CNV status of individual m6A regulatory gene, only the most frequent CNV type was investigated. The mRNA expression levels were calculated by RNA-Seq V2 RSEM release, which was accessed in the University of California Santa Cruz Xena data hub (UCSC Xena^2^). The immunohistochemistry (IHC) analysis of normal liver and HCC tissues were obtained from The Human Protein Atlas (HPA) database^3^.

### METTL16 mRNA Expression Analysis by Quantitative Real-Time PCR (qRT-PCR)

A commercial HCC cDNA microarray was purchased from Outdo biotech (Shanghai, China), which contains the cDNA of 66 HCC tissues and 21 matched para-cancerous tissues. The cDNA was amplified using SYBR-Green PCR Master Mix (TAKARA) on a QuantStudio 5 system (ABI, United States) under the conditions as follows: 95°C for 10 min, followed by 40 cycles of 95°C for 15 s and 60°C for 1 min. The relative expression level of METTL16 mRNA was calculated by 2^–Δ^ ^Δ^ ^*Ct*^ method using ACTB as internal control. The specific primers were as follows: METTL16-Forward: 5′- ATGTTGCGGGGTTGGTAT GA-3′; METTL16-Reverse: 5′- TGACGGAGGCAAAGCAGA TT-3′; ACTB-Forward: 5′- GAAGAGCTACGAGCTGCCTGA-3′; ACTB-Reverse: 5′- CAGACAGCACTGTGTTGGCG-3′.

### Gene Set Enrichment Analysis (GSEA)

The GESA-3.0.jar software was downloaded from the website of Broad Institute and ran under the support of Java 8.0 (Subramanian et al., 2005). In this study, the TCGA HCC cases were divided into two group (High and Low) according to the METTL16 expression level. Finally, 20531 genes were involved in the enrichment process. The KEGG gene sets (c2.cp.kegg.v6.2.symbols.gmt) were used in this study. Gene sets with normalized *p*-value < 0.05 and the false discovery rate (FDR) < 0.25 were considered to be significantly enriched.

### Statistical Analysis

All the data were analyzed by SPSS 17.0 (IBM, Chicago, IL, United States) and GraphPad Prism 6.0 (GraphPad Software, La Jolla, CA, United States). We used chi-square or Fisher’s exact test to analyze the association between the CNVs of m6A regulatory genes and clinicopathological characteristics. A one-way ANOVA test was used to compare the mRNA expression levels between groups of different CNV patterns. The Kaplan-Meier curve and log-rank test were performed to evaluate the prognostic values of m6A regulatory genes’ alteration. Cox proportional hazard regression model was performed to determine the prognostic values of various clinical and molecular characteristics. A *p*-value < 0.05 was considered statistically significant.

## Results

### Mutations and CNVs of m6A Regulatory Genes in HCC Patients

**FIGURE 1:**
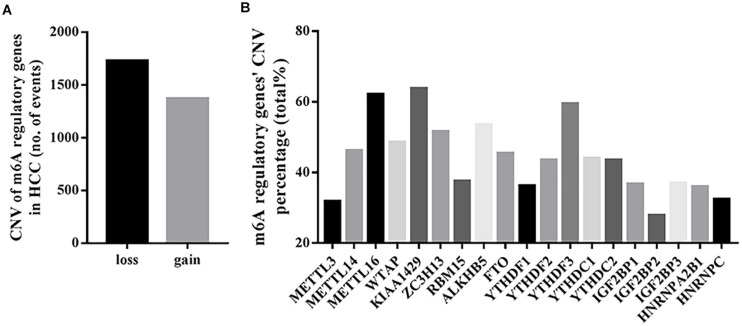
CNVs of m6A regulatory genes in HCC. **(A)** Events of copy number loss or gain of m6A regulatory genes in HCC samples. **(B)** Percentage of HCC samples with CNVs of m6A regulatory genes according to TCGA database.

Among the 373 cases with mutation data, mutations of m6A regulatory genes were found merely in 40 independent samples (Supplementary Table S1), while the CNVs were frequently observed in 370 HCC samples with CNV data. As shown in Table 1 and Figure 1A, a total of 3099 CNV events were found in 19 m6A regulatory genes. Among them, 1727 CNVs led to loss of copy number, and 1372 CNVs led to gain of copy number. All the writer and eraser genes except for KIAA1429 tend to loss of copy number, while most of the copy number gain events occurred in reader genes including YTHDF1, YTHDF3, YTHDC2, IGF2BP1, IGF2BP2, IGF2BP3, and HNRNPA2B1.

**TABLE 1:** The CNV patterns of m6A regulatory genes in HCC samples (*n* = 370).

		**Diploid**	**Deep deletion**	**Shallow deletion**	**Copy number gain**	**Amplification**	**CNV sum**	**Percentage**
**Writer**	**METTL3**	252		78	37	3	118	31.89%
	**METTL14**	199	1	160	10		171	46.21%
	**METTL16**	140	8	203	19		230	62.16%
	**WTAP**	190	7	132	40	1	180	48.65%
	**KIAA1429**	134	1	15	168	52	236	63.78%
	**ZC3H13**	179	8	170	13		191	51.62%
	**RBM15**	231		86	51	2	139	37.57%
**Eraser**	**ALKBH5**	172	4	156	35	3	198	53.51%
	**FTO**	202	4	143	21		168	45.41%
**Reader**	**YTHDF1**	236		8	119	7	134	36.22%
	**YTHDF2**	209	4	136	21		161	43.51%
	**YTHDF3**	150		30	159	31	220	59.46%
	**YTHDC1**	207		146	13	4	163	44.05%
	**YTHDC2**	209		46	112	3	161	43.51%
	**IGF2BP1**	234		27	102	7	136	36.76%
	**IGF2BP2**	267		43	52	8	103	27.84%
	**IGF2BP3**	233		16	116	5	137	37.03%
	**HNRNPA2B1**	237		16	113	4	133	35.95%
	**HNRNPC**	250		79	36	5	120	32.43%
	**TP53**	132	9	216	13		238	64.32%

*The CNV status was defined by GISTIC, including Deep deletion (homozygous deletion), Shallow deletion (heterozygous deletion), Diploid (normal copy number), Copy number gain (low level gain), and Amplification (high level amplification).*

In detail, the m6A writer gene KIAA1429 had the most frequent CNV events (236/370, 63.78%), followed by METTL16 (230/370, 62.16%) (Figure 1B), implying the important role of m6A writer genes in the process of m6A modification in HCC. Furthermore, we also observed frequent mutations (115/373, 30.83%) and CNVs (238/370, 64.32%) of TP53 (Table 1), which was in line with published literature (Nault et al., 2020).

### Alterations of m6A Regulatory Genes Were Associated With Clinicopathological and Molecular Features

We evaluated the relationship between the alterations (mutations and/or CNVs) of m6A regulatory genes and the clinicopathological features of HCC patients. The result showed that, as a group, the alterations of m6A regulatory genes were only associated with gender (*p* = 0.0005) (Table 2). Given the fact that TP53 play pivotal roles in the pathogenesis of HCC, we then evaluated whether the variation of m6A regulatory genes was associated with the alteration of TP53. As expected, the alterations of m6A regulatory genes were significantly associated with TP53 alteration (*p* = 0.0114) (Table 2).

**TABLE 2:** Clinical and molecular characteristics of TCGA HCC patients according to the mutation/CNV status of m6A regulatory genes.

		**With mutation and/or CNV**	**Without mutation or CNV**	***P*-value**
Age	≤60	65	110	0.8699
	>60	69	121	
Gender	male	106	142	**0.0005**
	female	28	89	
Histological grade	G1	13	37	0.3807
	G2	66	111	
	G3	48	72	
	G4	5	8	
Pathological stage	I	66	106	0.4808
	II	35	50	
	III	24	56	
	IV	2	3	
T stage	T1	69	113	0.6385
	T2	36	57	
	T3	24	51	
	T4	3	9	
	Tx	1	0	
N stage	N0	93	158	0.3010
	N1	0	3	
	Nx	40	70	
M stage	M0	98	165	0.5584
	M1	2	1	
	Mx	34	65	
TP53	alteration	55	65	**0.0114**
	wt	79	166	

*With mutation and/or CNV: cases have mutation or CNV or mutation + CNV. Without mutation or CNV: cases with neither mutation nor CNV. Ambiguous variables (Tx, Nx, and Mx) were excluded from chi-square test or Fisher’s exact test. Significant *P* values are in bold.*

We further determined whether the CNVs of individual m6A regulatory gene was associated with the clinicopathological and molecular features. The results showed that the CNVs of most m6A regulatory genes were closely related to the clinicopathological and molecular features (Supplementary Tables S2–S20). In detail, deep or shallow deletion of METTL14, METTL16, ZC3H13, RBM15, ALKBH5, FTO, YTHDC1, and HNRNPC, and copy number gain of METTL3, KIAA1429, YTHDF1, YTHDF3, IGF2BP1, and IGF2BP2 were significantly associated with higher histological grade and/or TNM stage. Moreover, deep or shallow deletion of METTL14, METTL16, ZC3H13, ALKBH5, FTO, YTHDF2, YTHDC1, and HNRNPC, and copy number gain of IGF2BP1 and IGF2BP2 were significantly associated with the presence of TP53 alteration, which was consistent with our findings in m6A regulatory genes overall.

**FIGURE 2:**
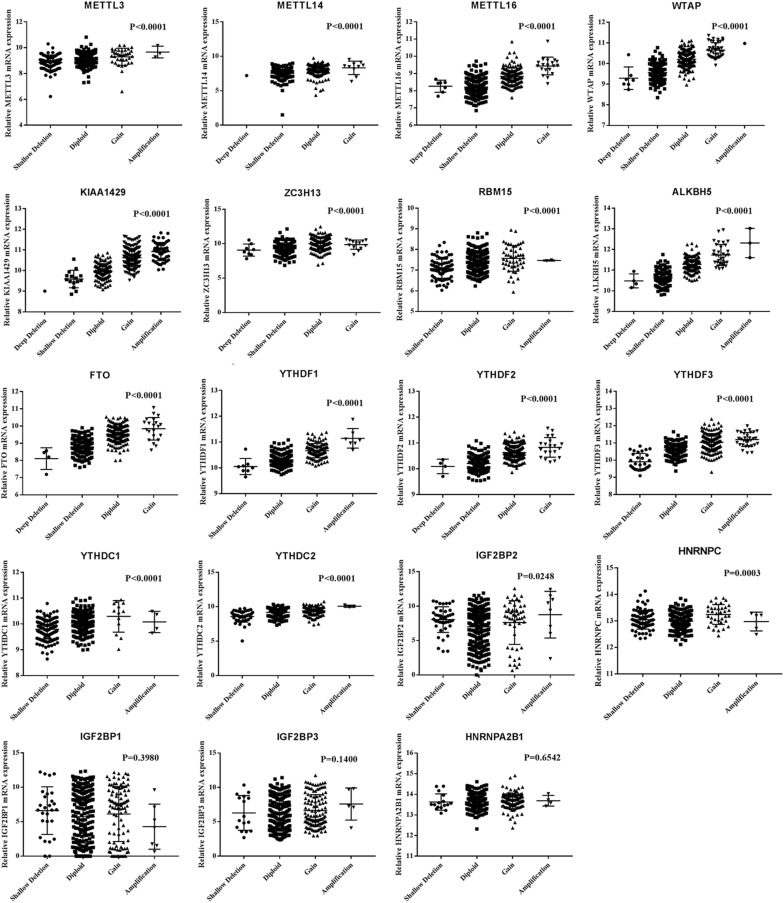
MRNA expression levels of m6A regulatory genes in different CNV patterns.

Next, we evaluated the effect of alterations in m6A regulatory genes on mRNA expression level. As expected, the mRNA levels were significantly associated with different CNV patterns. For all the m6A writers and erasers, and most of the m6A readers, deep or shallow deletions resulted in lower mRNA expression, while amplifications or copy number gains were related to higher mRNA expression (Figure 2).

### Association Between Alterations of m6A Regulatory Genes and Survival of HCC Patients

**FIGURE 3:**
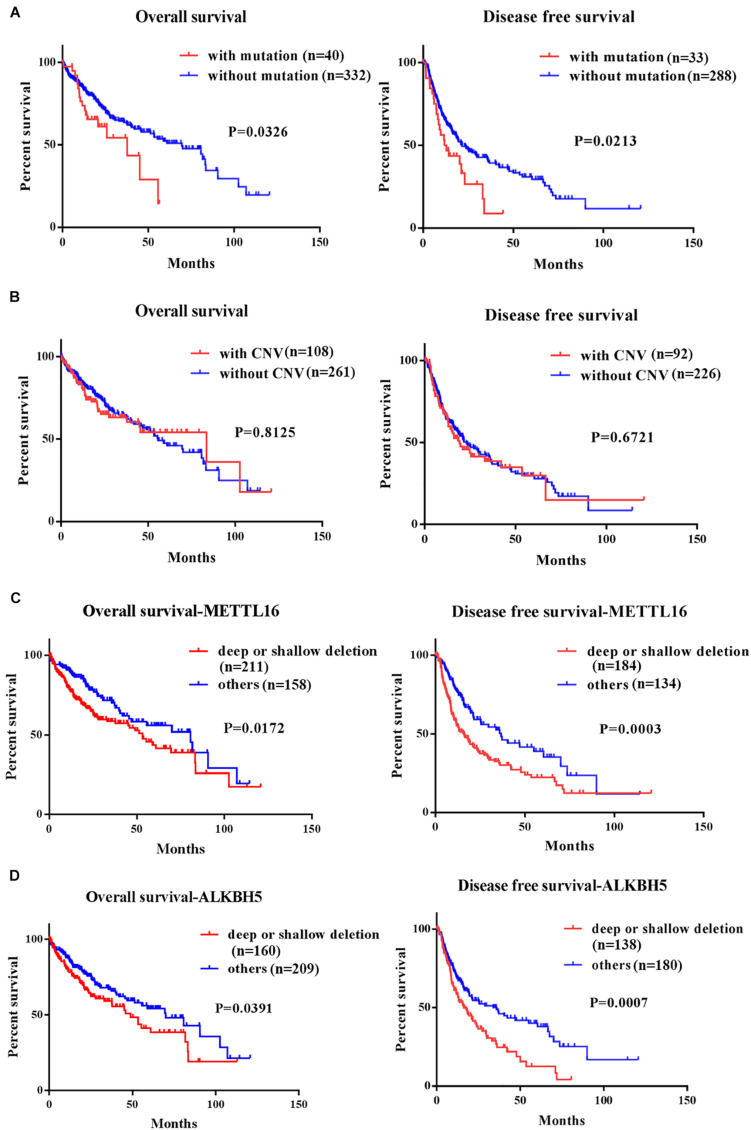
Kaplan-Meier curves for overall and disease free survival of HCC patients in TCGA according to the presence or absence of **(A)** mutation of m6A regulatory genes, **(B)** CNV of m6A regulatory genes, **(C)** deep/shallow deletion of METTL16, and **(D)** deep/shallow deletion of ALKHB5.

We performed Kaplan-Meier analysis to investigate the impact of genetic alterations in m6A regulators on overall (OS) and disease-free survival (DFS) in patients with HCC. As a group, patients with any mutation of the m6A regulatory genes had worse OS (*P* = 0.0326) and DFS (*P* = 0.0213) (Figure 3A). However, patients with or without CNVs of m6A regulatory genes didn’t have any correlation with OS and DFS (Figure 3B). Further separate analysis of the 19 genes revealed that patients with deletion of METTL16 or ALKBH5 had worse OS and DFS (Figures 3C,D), while no significant association between CNVs and survival was found for other m6A regulatory genes (Supplementary Figures S1, S2). To determine the prognostic values of METTL16 and ALKBH5, we performed Multivariate Cox regression analyses. The result indicated that deletion of METTL16 was an independent risk factor of DFS for HCC patients (Table 3).

**TABLE 3:** Univariate and Multivariate Cox regression analysis of METTL16 and ALKBH5 for HCC patients’ OS and DFS.

	**OS**				**DFS**			
**Variables**	**Univariate**		**Multivariate**		**Univariate**		**Multivariate**	
	**HR(95%CI)**	***P*-value**	**HR(95%CI)**	***P*-value**	**HR(95%CI)**	***P*-value**	**HR(95%CI)**	***P*-value**
Age (>60 vs. ≤60)	1.246(0.877–1.769)	0.219			0.956(0.709–1.290)	0.768		
Gender (male vs. female)	1.243(0.870–1.775)	0.232			1.107(0.804–1.525)	0.533		
Grade (3–4 vs. 1–2)	1.073(0.746–1.542)	0.704			1.152(0.846–1.569)	0.369		
Stage (III–IV vs. I–II)	2.327(1.598–3.390)	**<0.0001**	1.833(0.244–13.763)	0.556	2.087(1.489–2.925)	**<0.0001**	1.836(1.239–2.721)	**0**.**002**
T (T3–T4 vs. T1–T2)	2.427(1.699–3.467)	**<0.0001**	2.507(1.619–3.883)	**<0.0001**	2.049(1.475–2.845)	**<0.0001**	0.987(0.227–4.286)	0.987
N (N1 vs. N0)	1.252(0.174–9.028)	0.824			0.884(1.123–6.337)	0.902		
M (M1 vs. M0)	4.183(1.313–13.322)	**0.015**	2.079(0.623–6.940)	0.234	4.945(1.548–15.796)	**0.007**	3.517(1.006–11.602)	**0**.**039**
TP53 (Alteration vs. WT)	1.517(1.049–2.193)	**0.027**	1.313(0.807–2.317)	0.237	1.587(1.163–2.165)	**0.004**	1.201(0.794–1.815)	0.385
METTL16 (Deletion vs. others)	1.541(1.077–2.207)	**0.018**	1.583(0.999–2.510)	0.051	1.773(1.294–2.428)	**<0.0001**	1.979(1.342–2.918)	**0**.**001**
ALKBH5 (Deletion vs. others)	1.438(1.016–2.034)	**0.040**	1.135(0.679–1.897)	0.629	1.676(1.238–2.268)	**0.001**	1.067(0.675–1.687)	0.781

*HR, hazard ratio; CI, confidence interval. Significant *P* values are in bold.*

### The Expression of METTL16 mRNA Was Decreased in HCC and Was Associated With Poor Prognosis

**FIGURE 4:**
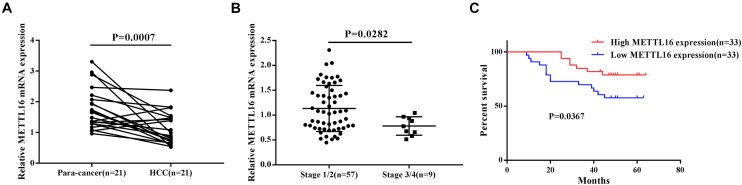
METTL16 was downregulated in HCC and was associated with poor prognosis. **(A)** The mRNA level of METTL16 in 21 pairs of HCC tissues and their adjacent para-cancerous tissues. **(B)** The mRNA level of METTL16 in HCC patients with different clinical stage. **(C)** Kaplan-Meier curve for overall survival of HCC patients according to the high or low level of METTL16.

To confirm our conclusions above, we measured the mRNA level of METTL16 in 66 HCC tissues and 21 para-cancerous tissues by RT-PCR. The results showed that METTL16 mRNA was significantly downregulated in HCC tissues compared with matched para-cancerous tissues (Figure 4A, *P* = 0.0007). Moreover, we confirmed the downregulation of METTL16 protein in HCC using the IHC staining results from the Human Protein Atlas database (Supplementary Figure S3). By searching Oncomine database, we also found a copy number loss of METTL16 gene in Guichard’s HCC cohort (Supplementary Figure S4). Then we evaluated the correlation between METTL16 mRNA expression and the clinicopathological features of HCC patients. Although no significant correlations were observed between METTL16 expression and clinicopathological features such as age, gender, pathological grade, clinical stage, and tumor size (Supplementary Table S21), we found a lower level of METTL16 in patients with stage 3/4 than that in patients with stage 1/2 (Figure 4B, *P* = 0.0282). In addition, Kaplan-Meier analysis indicated that lower METTL16 mRNA expression in HCC tissues was significantly associated with a poor OS (Figure 4C, *P* = 0.0367). Moreover, We also investigated the association of METTL16 mRNA expression and patient survival in TCGA HCC dataset. We found a trend, though not significant, for poorer OS of patients with lower METTL16 expression levels. The mean survival time of low expression cohort and high expression cohort were 54.1 months and 81.9 months, respectively (Supplementary Figure S5). Taken together, our RT-PCR results are consistent with the findings of bioinformatic analyses.

### Functional Enrichment Analysis of METTL16

**FIGURE 5:**
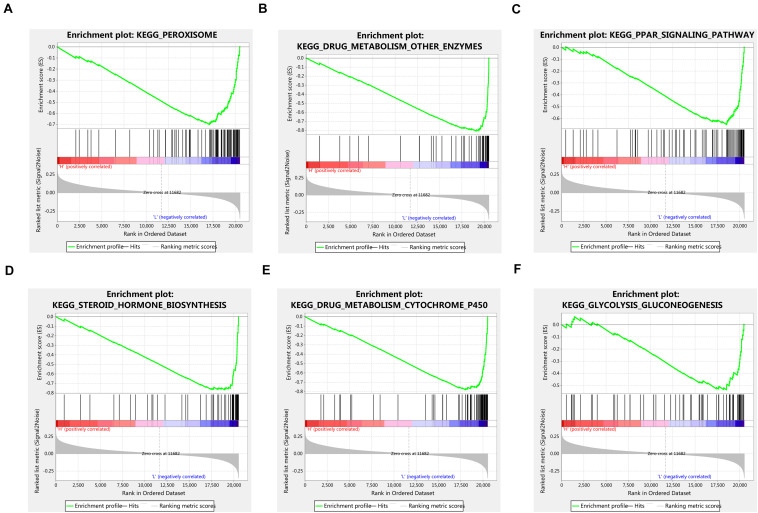
GSEA analysis indicated that low expression of METTL16 was significantly correlated with **(A)** peroxisome, **(B)** drug metabolism other enzymes, **(C)** PPAR signaling pathway, **(D)** steroid hormone biosynthesis, **(E)** drug metabolism cytochrome p450, and **(F)** glycolysis gluconeogenesis pathways in TCGA HCC dataset. The ES indicates the degree to which the specified gene sets are overrepresented at the top/bottom of a ranked list of total protein-coding genes. For each enrichment plot, top portion: running ES for the specified gene set; middle portion: where the member of gene set appears in the ranked list of all protein-coding genes; bottom portion: value of the ranking metric as a measurement of gene’s correlation with METTL16.

Given the important role of METTL16 in m6A modification process and the interesting results we found in this study, we determined to explore the potential function of METTL16 in HCC. The gene set enrichment analysis (GSEA) were performed using data from TCGA database. The result suggested that multiple metabolic pathways including peroxisome, drug metabolism, PPAR signaling pathway, steroid hormone biosynthesis, and glycolysis gluconeogenesis, were significantly enriched in the group with low METTL16 expression (Figure 5 and Table 4). Moreover, we found that several genes related to the above pathways were significantly upregulated in HCC, which partially validated the results of GESA (Supplementary Figure S6). Further studies are needed to illustrate the functional targets of METTL16 in the pathogenesis of HCC.

**TABLE 4:** Gene sets enrichment of low METTL16 mRNA expression level in the TCGA HCC dataset.

**KEGG Pathways**	**ES**	**NES**	**Nominal *P*-value**	**FDR *q*-value**
KEGG_PEROXISOME	−0.70	−2.04	< 0.001	0.044
KEGG_DRUG_METABOLISM_OTHER_ENZYMES	−0.81	−1.86	< 0.001	0.062
KEGG_PPAR_SIGNALING_PATHWAY	−0.65	−1.85	0.002	0.055
KEGG_STEROID_HORMONE_BIOSYNTHESIS	−0.77	−1.73	0.004	0.110
KEGG_DRUG_METABOLISM_CYTOCHROME_P450	−0.78	−1.72	< 0.001	0.087
KEGG_GLYCOLYSIS_GLUCONEOGENESIS	−0.54	−1.70	0.002	0.076

*ES, Enrichment Score; NES, Normalized Enrichment Score. The NES is the enrichment score for the gene set after it has been normalized by gene numbers. The NES is calculated by dividing the actual ES score by the mean ES of all random combinations of gene sets.*

## Discussion

Accumulating studies have reported the important role of m6A modification in tumorigenesis and progression (Wang et al., 2018). However, the alterations and biological role of m6A modification remain elusive in HCC. In this study, we analyzed the alterations of m6A regulatory genes in HCC, and found that the frequency of the alterations was much higher than that reported in acute myeloid leukemia (AML) (Kwok et al., 2017) and clear cell renal cell carcinoma (ccRCC) (Zhou et al., 2019), implying that dysregulation of m6A might play a more important role in HCC tumorigenesis. Among the 19 m6A regulatory genes, KIAA1429 and METTL16 had the most frequent CNV events, indicating the critical role of m6A writer genes in m6A modification of HCC. Moreover, we found that the alterations of KIAA1429 and YTHDF1 tend to gain of copy number, while METTL14 and YTHDF2 tend to loss of copy number. These findings were consistent with previous studies, which reported the upregulation of KIAA1429 (Lan et al., 2019) and YTHDF1 (Zhao et al., 2018), and downregulation of METTL14 (Ma et al., 2017) and YTHDF2 (Hou et al., 2019) in HCC.

Previous studies have suggested that the risk of developing HCC in males is higher compared with that in females (Yeh and Chen, 2010; Liu et al., 2019). Irrespective of the etiology, the morbidity of liver cancer in males is 2–4-fold higher compared with that in females (El-Serag, 2011). In this study, we found that the mutation or CNV events of m6A related genes were more common in male than that in female patients with HCC, suggesting a possible role for m6A modification in sex disparity. Moreover, we found that, for most of the m6A regulatory genes, their CNVs were significantly associated with histological grade or TNM stage, prompting that dysregulation of m6A methylation were closely correlated with HCC development. In addition, we observed the alteration of m6A regulatory genes was significantly associated with the alteration of TP53, an important tumor suppressor genes in HCC. It has been reported that silencing METTL3 in a HCC cell line HepG2 affects the expression and alternative splicing pattern of more than 20 genes involved in the TP53 signaling pathway (Dominissini et al., 2012). Thus, genetic alterations of m6A regulators may cooperate with TP53 signaling pathway in the pathogenesis of HCC.

We also evaluated the effect of m6A regulators’ alterations on the survival of HCC patients. We found that patients with any mutation of m6A regulatory genes had a worse OS and DFS. Further separate analysis showed that only METTL16 and ALKBH5 among the 19 regulators were associated with survival of HCC patients. Moreover, the result from the Multivariate Cox regression analysis indicated that copy number loss of METTL16 was an independent risk factor for DFS. It has been reported that several m6A regulators including METTL3 (Chen et al., 2018), METTL14 (Ma et al., 2017), WTAP (Chen et al., 2019), KIAA1429 (Lan et al., 2019), and YTHDF2 (Hou et al., 2019), play critical roles in the carcinogenesis and progression of HCC. Here, we report for the first time that the alterations of METTL16 and ALKBH5 are closely associated with worse clinical characteristics including survival, and loss of METTL16 is an independent risk factor of DFS, suggesting their potential roles in HCC carcinogenesis and progression.

Recently, the mRNA methylation complex containing METTL3, METTL14, and WTAP has been the subject of intense study in human cancers (He et al., 2019). METTL16 is an independent RNA m6A methyltransferase that can methylate both coding and non-coding RNA, but its biological role is rarely studied. Although many potential RNA targets of METTL16 have been identified by high throughput method, only a few, including U6 snRNA (Warda et al., 2017), Long non-coding RNA MALAT1 (Brown et al., 2016), and mRNA MAT2A (Pendleton et al., 2017) have been verified. The MAT2A gene encodes a methionine adenosyltransferase that catalyzes the production of S-adenosylmethionine, a key substrate required for RNA methylation. It is reported that METTL16 could induce splicing of MAT2A mRNA, which increases the stability and translation of MAT2A mRNA (Pendleton et al., 2017). Thus, loss of METTL16 may result in a transcriptome-wide loss of m6A methylation by modulating S-adenosylmethionine production (Koh et al., 2019). In this study, we observed a copy number loss with down-regulation of METTL16, which might explain the previous findings that the m6A level of total RNA was decreased in HCC (Ma et al., 2017; Hou et al., 2019).

Metabolic reprogramming has been recognized as a hallmark of cancer (Pavlova and Thompson, 2016). The cancer cells reprogram their metabolism to support their rapid proliferation. A previous TCGA analysis highlighted the critical role of metabolic reprogramming in the progression of HCC (Cancer Genome Atlas Research Network, 2017). In the present study, to explore the potential biological role of METTL16 in HCC, we performed GSEA analysis in the TCGA cohort and found that low METTL16 level was associated with the activation of multiple metabolic pathways, implying a potential role of METTL16 in metabolic reprogramming.

In conclusion, our present study determined the alterations of 19 m6A regulatory genes in HCC and their association with clinicopathological features including survival. We found significant associations between the genetic alterations and clinicopathological features as well as TP53 alteration. Notably, we found that loss of METTL16 predicted a worse survival and was an independent risk factor of DFS for HCC patients. Our study highlight METTL16 as a potential prognostic biomarker as well as potential target for HCC treatment. These findings provide a new insight into the underlying mechanism of m6A modification in tumorigenesis and development of HCC.

## Data Availability Statement

The datasets generated for this study can be found in The Cancer Genome Atlas (https://portal.gdc.cancer.gov/).

## Ethics Statement

Written informed consent was obtained from the individual(s) for the publication of any potentially identifiable images or data included in this article.

## Author Contributions

PW and CZ conceived and designed the study and wrote the initial draft of the manuscript. PW, XW, and LZ collected and analyzed the data. All authors contributed to the article and approved the submitted version.

## Conflict of Interest

The authors declare that the research was conducted in the absence of any commercial or financial relationships that could be construed as a potential conflict of interest.

## Footnotes

1 http://www.cbioportal.org

2 https://xena.ucsc.edu/

3 https://www.proteinatlas.org
