# Supplementary material for: Gene Signatures and Prognostic Values of m6A Regulators in Hepatocellular Carcinoma
Source: Front Genet. 2020 Oct 2;11:540186. doi: 10.3389/fgene.2020.540186 (PMC7567013; doi:10.3389/fgene.2020.540186)
Supplement: Supplementary file 1 [file Data_Sheet_1.docx]

Supplementary Tables

**Table S1** Mutations of m6A regulatory genes in 373 HCC patients

| **HCC**  **sample ID** | **METTL16** | **WTAP** | **VIRMA** | **ZC3H13** | **RBM15** | **ALKBH5** | **FTO** | **YTHDF1** |
| --- | --- | --- | --- | --- | --- | --- | --- | --- |
| [TCGA-EP-A2KA](https://www.cbioportal.org/patient?studyId=lihc_tcga&caseId=TCGA-EP-A2KA) | K385R |  |  |  |  |  |  |  |
| [TCGA-DD-AAE4](https://www.cbioportal.org/patient?studyId=lihc_tcga&caseId=TCGA-DD-AAE4) | G110S |  |  |  |  |  |  |  |
| [TCGA-DD-AAEA](http://www.cbioportal.org/patient?studyId=lihc_tcga&caseId=TCGA-DD-AAEA) |  | I208T |  |  |  |  |  |  |
| [TCGA-DD-A39Y](https://www.cbioportal.org/patient?studyId=lihc_tcga&caseId=TCGA-DD-A39Y) |  |  | N821Ifs*8 |  |  |  |  |  |
| [TCGA-DD-AAC8](https://www.cbioportal.org/patient?studyId=lihc_tcga&caseId=TCGA-DD-AAC8) |  |  | K461* |  |  |  |  |  |
| [TCGA-5R-AA1C](https://www.cbioportal.org/patient?studyId=lihc_tcga&caseId=TCGA-5R-AA1C) |  |  | Q68* |  |  |  |  |  |
| [TCGA-DD-AACX](https://www.cbioportal.org/patient?studyId=lihc_tcga&caseId=TCGA-DD-AACX) |  |  | X1462_splice |  |  |  |  |  |
| [TCGA-G3-A5SL](https://www.cbioportal.org/patient?studyId=lihc_tcga&caseId=TCGA-G3-A5SL) |  |  | N1088S  D193N |  |  |  |  |  |
| [TCGA-HP-A5N0](https://www.cbioportal.org/patient?studyId=lihc_tcga&caseId=TCGA-HP-A5N0) |  |  | L1115I |  |  |  |  |  |
| [TCGA-UB-A7MB](https://www.cbioportal.org/patient?studyId=lihc_tcga&caseId=TCGA-UB-A7MB) |  |  | P1767T |  |  |  |  |  |
| [TCGA-BD-A3ER](https://www.cbioportal.org/patient?studyId=lihc_tcga&caseId=TCGA-BD-A3ER) |  |  |  | S19N |  |  |  |  |
| [TCGA-DD-AADO](https://www.cbioportal.org/patient?studyId=lihc_tcga&caseId=TCGA-DD-AADO) |  |  |  | R1271* |  |  |  |  |
| [TCGA-DD-AAVX](https://www.cbioportal.org/patient?studyId=lihc_tcga&caseId=TCGA-DD-AAVX) |  |  |  | R1325L |  |  |  |  |
| [TCGA-DD-AA3A](https://www.cbioportal.org/patient?studyId=lihc_tcga&caseId=TCGA-DD-AA3A) |  |  |  |  | S64W |  |  |  |
| [TCGA-G3-A5SK](https://www.cbioportal.org/patient?studyId=lihc_tcga&caseId=TCGA-G3-A5SK) |  |  |  |  |  | D332V |  |  |
| [TCGA-DD-A1EF](http://www.cbioportal.org/patient?studyId=lihc_tcga&caseId=TCGA-DD-A1EF) |  |  |  |  |  |  | H261Q |  |
| [TCGA-G3-A5SK](http://www.cbioportal.org/patient?studyId=lihc_tcga&caseId=TCGA-G3-A5SK) |  |  |  |  |  |  |  |  |
| [TCGA-4R-AA8I](http://www.cbioportal.org/patient?studyId=lihc_tcga&caseId=TCGA-4R-AA8I) |  |  |  |  |  |  |  | D401G |
| [TCGA-DD-A1EE](http://www.cbioportal.org/patient?studyId=lihc_tcga&caseId=TCGA-DD-A1EE) |  |  |  |  |  |  |  | G65R |
| [TCGA-ES-A2HT](http://www.cbioportal.org/patient?studyId=lihc_tcga&caseId=TCGA-ES-A2HT) |  |  |  |  |  |  |  | D80G |

**Table S1** (continued)

| **HCC**  **sample ID** | **YTHDC1** | **YTHDC2** | **IGF2BP1** | **IGF2BP2** | **IGF2BP3** | **HNRNPA2B1** | **HNRNPC** |
| --- | --- | --- | --- | --- | --- | --- | --- |
| [TCGA-XR-A8TG](http://www.cbioportal.org/patient?studyId=lihc_tcga&caseId=TCGA-XR-A8TG) | R711L |  |  |  |  |  |  |
| [TCGA-2Y-A9H9](http://www.cbioportal.org/patient?studyId=lihc_tcga&caseId=TCGA-2Y-A9H9) | F492L |  |  |  |  |  |  |
| [TCGA-G3-A3CJ](http://www.cbioportal.org/patient?studyId=lihc_tcga&caseId=TCGA-G3-A3CJ) | K408Nfs*27 |  |  |  |  |  |  |
| [TCGA-DD-AAE8](http://www.cbioportal.org/patient?studyId=lihc_tcga&caseId=TCGA-DD-AAE8) | Y725S |  |  |  |  |  |  |
| [TCGA-CC-A3M9](http://www.cbioportal.org/patient?studyId=lihc_tcga&caseId=TCGA-CC-A3M9) |  | M795T |  |  |  |  |  |
| [TCGA-CC-A7IK](http://www.cbioportal.org/patient?studyId=lihc_tcga&caseId=TCGA-CC-A7IK) |  | C25R |  |  |  |  |  |
| [TCGA-DD-A119](http://www.cbioportal.org/patient?studyId=lihc_tcga&caseId=TCGA-DD-A119) |  | R1347G |  |  |  |  |  |
| [TCGA-LG-A9QC](http://www.cbioportal.org/patient?studyId=lihc_tcga&caseId=TCGA-LG-A9QC) |  | I734M |  |  |  |  |  |
| [TCGA-DD-AAD2](http://www.cbioportal.org/patient?studyId=lihc_tcga&caseId=TCGA-DD-AAD2) |  | C885F |  |  |  |  |  |
| [TCGA-DD-A3A0](http://www.cbioportal.org/patient?studyId=lihc_tcga&caseId=TCGA-DD-A3A0) |  | L1135Yfs*4  S1335Lfs*31 |  |  |  |  |  |
| [TCGA-CC-A3MB](http://www.cbioportal.org/patient?studyId=lihc_tcga&caseId=TCGA-CC-A3MB) |  | A1025V |  |  |  |  |  |
| [TCGA-ED-A459](http://www.cbioportal.org/patient?studyId=lihc_tcga&caseId=TCGA-ED-A459) |  | I616F |  |  |  |  |  |
| [TCGA-BC-A216](https://www.cbioportal.org/patient?studyId=lihc_tcga&caseId=TCGA-BC-A216) |  |  | X273_splice |  |  |  |  |
| [TCGA-DD-AAC8](https://www.cbioportal.org/patient?studyId=lihc_tcga&caseId=TCGA-DD-AAC8) |  |  |  | S369C |  |  |  |
| [TCGA-DD-A1EG](https://www.cbioportal.org/patient?studyId=lihc_tcga&caseId=TCGA-DD-A1EG) |  |  |  |  | A239Lfs*33 |  |  |
| [TCGA-CC-A7II](https://www.cbioportal.org/patient?studyId=lihc_tcga&caseId=TCGA-CC-A7II) |  |  |  |  | K36* |  |  |
| [TCGA-ED-A7PZ](https://www.cbioportal.org/patient?studyId=lihc_tcga&caseId=TCGA-ED-A7PZ) |  |  |  |  | L196M |  |  |
| [TCGA-DD-AAEH](https://www.cbioportal.org/patient?studyId=lihc_tcga&caseId=TCGA-DD-AAEH) |  |  |  |  |  | S311del |  |
| [TCGA-ED-A66Y](https://www.cbioportal.org/patient?studyId=lihc_tcga&caseId=TCGA-ED-A66Y) |  |  |  |  |  | P274H |  |
| [TCGA-DD-AAE3](https://www.cbioportal.org/patient?studyId=lihc_tcga&caseId=TCGA-DD-AAE3) |  |  |  |  |  |  | M74V |
| [TCGA-CC-A5UE](https://www.cbioportal.org/patient?studyId=lihc_tcga&caseId=TCGA-CC-A5UE) |  |  |  |  |  |  | E287* |
| [TCGA-DD-A39X](https://www.cbioportal.org/patient?studyId=lihc_tcga&caseId=TCGA-DD-A39X) |  |  |  |  |  |  | L84R |

**Table S2 Clinical and molecular characteristics of TCGA HCC patients with an amplification or copy number gain of METTL3**

|  |  | **Amplification or copy number gain of METTL3** | | P |
| --- | --- | --- | --- | --- |
|  |  | yes | no |  |
| Age | <60 | 17 | 158 | 0.5088 |
|  | >60 | 23 | 171 |  |
| Gender | male | 24 | 228 | 0.2440 |
|  | female | 16 | 102 |  |
| Histological grade | G1 | 3 | 49 | **0.0382** |
|  | G2 | 16 | 161 |  |
|  | G3 | 21 | 102 |  |
|  | G4 | 0 | 13 |  |
| Pathological stage | I | 18 | 155 | 0.7248 |
|  | II | 8 | 79 |  |
|  | III | 11 | 70 |  |
|  | IV | 1 | 4 |  |
| T stage | T1 | 20 | 163 | 0.8905 |
|  | T2 | 9 | 86 |  |
|  | T3 | 10 | 67 |  |
|  | T4 | 1 | 11 |  |
|  | Tx | 0 | 1 |  |
| N stage | N0 | 27 | 227 | 0.2935 |
|  | N1 | 1 | 2 |  |
|  | Nx | 11 | 101 |  |
| M stage | M0 | 27 | 239 | 0.3563 |
|  | M1 | 1 | 3 |  |
|  | Mx | 12 | 88 |  |
| TP53 | alteration | 16 | 104 | 0.3059 |
|  | wt | 24 | 221 |  |

**Table S3 Clinical and molecular characteristics of TCGA HCC patients with a deep or shallow deletion of METTL14**

|  |  | **Deep or shallow deletion of METTL14** | | P |
| --- | --- | --- | --- | --- |
|  |  | yes | no |  |
| Age | <60 | 91 | 84 | **0.0021** |
|  | >60 | 70 | 124 |  |
| Gender | male | 113 | 139 | 0.4516 |
|  | female | 48 | 70 |  |
| Histological grade | G1 | 15 | 37 | **0.0009** |
|  | G2 | 68 | 109 |  |
|  | G3 | 71 | 52 |  |
|  | G4 | 6 | 7 |  |
| Pathological stage | I | 70 | 103 | 0.4001 |
|  | II | 44 | 43 |  |
|  | III | 35 | 46 |  |
|  | IV | 3 | 2 |  |
| T stage | T1 | 73 | 110 | 0.3643 |
|  | T2 | 47 | 48 |  |
|  | T3 | 36 | 41 |  |
|  | T4 | 4 | 8 |  |
|  | Tx | 1 | 0 |  |
| N stage | N0 | 114 | 140 | >0.9999 |
|  | N1 | 1 | 2 |  |
|  | Nx | 45 | 67 |  |
| M stage | M0 | 128 | 138 | 0.3579 |
|  | M1 | 3 | 1 |  |
|  | Mx | 30 | 70 |  |
| TP53 | alteration | 84 | 36 | **<0.0001** |
|  | wt | 76 | 169 |  |

**Table S4 Clinical and molecular characteristics of TCGA HCC patients with a deep or shallow deletion of METTL16**

|  |  | **Deep or shallow deletion of METTL16** | | P |
| --- | --- | --- | --- | --- |
|  |  | yes | no |  |
| Age | <60 | 106 | 69 | 0.2113 |
|  | >60 | 105 | 89 |  |
| Gender | male | 146 | 106 | 0.6056 |
|  | female | 65 | 53 |  |
| Histological grade | G1 | 21 | 31 | **0.0113** |
|  | G2 | 102 | 75 |  |
|  | G3 | 76 | 47 |  |
|  | G4 | 11 | 2 |  |
| Pathological stage | I | 86 | 87 | **0.0354** |
|  | II | 57 | 30 |  |
|  | III | 51 | 30 |  |
|  | IV | 4 | 1 |  |
| T stage | T1 | 93 | 90 | 0.0667 |
|  | T2 | 63 | 32 |  |
|  | T3 | 47 | 30 |  |
|  | T4 | 8 | 4 |  |
|  | Tx | 0 | 1 |  |
| N stage | N0 | 149 | 105 | >0.9999 |
|  | N1 | 2 | 1 |  |
|  | Nx | 59 | 53 |  |
| M stage | M0 | 157 | 109 | 0.6479 |
|  | M1 | 3 | 1 |  |
|  | Mx | 51 | 49 |  |
| TP53 | alteration | 108 | 12 | **<0.0001** |
|  | wt | 102 | 143 |  |

**Table S5 Clinical and molecular characteristics of TCGA HCC patients with a deep or shallow deletion of WTAP**

|  |  | **Deep or shallow deletion of WTAP** | | P |
| --- | --- | --- | --- | --- |
|  |  | yes | no |  |
| Age | <60 | 56 | 119 | **0.0238** |
|  | >60 | 84 | 111 |  |
| Gender | male | 91 | 161 | 0.3979 |
|  | female | 48 | 70 |  |
| Histological grade | G1 | 12 | 40 | 0.1527 |
|  | G2 | 67 | 110 |  |
|  | G3 | 49 | 74 |  |
|  | G4 | 6 | 7 |  |
| Pathological stage | I | 60 | 113 | 0.3539 |
|  | II | 33 | 54 |  |
|  | III | 36 | 45 |  |
|  | IV | 3 | 2 |  |
| T stage | T1 | 64 | 119 | 0.7503 |
|  | T2 | 37 | 58 |  |
|  | T3 | 32 | 45 |  |
|  | T4 | 4 | 8 |  |
|  | Tx | 1 | 0 |  |
| N stage | N0 | 96 | 158 | 0.5598 |
|  | N1 | 2 | 1 |  |
|  | Nx | 40 | 72 |  |
| M stage | M0 | 107 | 159 | 0.3075 |
|  | M1 | 3 | 1 |  |
|  | Mx | 29 | 71 |  |
| TP53 | alteration | 41 | 79 | 0.3524 |
|  | wt | 96 | 149 |  |

**Table S6 Clinical and molecular characteristics of TCGA HCC patients with an amplification or copy number gain of KIAA1429**

|  |  | **Amplification or copy number gain of KIAA1429** | | P |
| --- | --- | --- | --- | --- |
|  |  | yes | no |  |
| Age | <60 | 113 | 62 | 0.0524 |
|  | >60 | 106 | 88 |  |
| Gender | male | 167 | 85 | **<0.0001** |
|  | female | 53 | 65 |  |
| Histological grade | G1 | 18 | 34 | **<0.0001** |
|  | G2 | 104 | 73 |  |
|  | G3 | 85 | 38 |  |
|  | G4 | 11 | 2 |  |
| Pathological stage | I | 104 | 69 | 0.3946 |
|  | II | 59 | 28 |  |
|  | III | 47 | 34 |  |
|  | IV | 2 | 3 |  |
| T stage | T1 | 106 | 77 | 0.3789 |
|  | T2 | 62 | 33 |  |
|  | T3 | 46 | 31 |  |
|  | T4 | 5 | 7 |  |
|  | Tx | 0 | 1 |  |
| N stage | N0 | 163 | 91 | **0.0479** |
|  | N1 | 0 | 3 |  |
|  | Nx | 57 | 55 |  |
| M stage | M0 | 167 | 99 | 0.6314 |
|  | M1 | 2 | 2 |  |
|  | Mx | 51 | 49 |  |
| TP53 | alteration | 75 | 45 | 0.3662 |
|  | wt | 141 | 104 |  |

**Table S7 Clinical and molecular characteristics of TCGA HCC patients with a deep or shallow deletion of ZC3H13**

|  |  | **Deep or shallow deletion of ZC3H13** | | P |
| --- | --- | --- | --- | --- |
|  |  | yes | no |  |
| Age | <60 | 93 | 82 | 0.0733 |
|  | >60 | 85 | 109 |  |
| Gender | male | 113 | 139 | 0.0661 |
|  | female | 65 | 53 |  |
| Histological grade | G1 | 24 | 28 | **0.0007** |
|  | G2 | 68 | 109 |  |
|  | G3 | 76 | 47 |  |
|  | G4 | 8 | 5 |  |
| Pathological stage | I | 77 | 96 | 0.5676 |
|  | II | 46 | 41 |  |
|  | III | 40 | 41 |  |
|  | IV | 3 | 2 |  |
| T stage | T1 | 82 | 101 | 0.0588 |
|  | T2 | 50 | 45 |  |
|  | T3 | 36 | 41 |  |
|  | T4 | 10 | 2 |  |
|  | Tx | 0 | 1 |  |
| N stage | N0 | 127 | 127 | >0.9999 |
|  | N1 | 2 | 1 |  |
|  | Nx | 48 | 64 |  |
| M stage | M0 | 130 | 136 | >0.9999 |
|  | M1 | 2 | 2 |  |
|  | Mx | 46 | 54 |  |
| TP53 | alteration | 77 | 43 | **<0.0001** |
|  | wt | 100 | 145 |  |

**Table S8 Clinical and molecular characteristics of TCGA HCC patients with a shallow deletion of RBM15**

|  |  | **Shallow deletion of RBM15** | | P |
| --- | --- | --- | --- | --- |
|  |  | yes | no |  |
| Age | <60 | 49 | 126 | **0.0428** |
|  | >60 | 37 | 157 |  |
| Gender | male | 55 | 197 | 0.3454 |
|  | female | 31 | 87 |  |
| Histological grade | G1 | 7 | 45 | **0.0051** |
|  | G2 | 35 | 142 |  |
|  | G3 | 42 | 80 |  |
|  | G4 | 2 | 11 |  |
| Pathological stage | I | 37 | 136 | 0.1733 |
|  | II | 24 | 63 |  |
|  | III | 18 | 63 |  |
|  | IV | 3 | 2 |  |
| T stage | T1 | 39 | 144 | 0.3830 |
|  | T2 | 25 | 70 |  |
|  | T3 | 21 | 56 |  |
|  | T4 | 1 | 11 |  |
|  | Tx | 0 | 1 |  |
| N stage | N0 | 60 | 194 | >0.9999 |
|  | N1 | 0 | 3 |  |
|  | Nx | 26 | 86 |  |
| M stage | M0 | 71 | 195 | 0.0640 |
|  | M1 | 3 | 1 |  |
|  | Mx | 12 | 88 |  |
| TP53 | alteration | 25 | 95 | 0.4886 |
|  | wt | 59 | 186 |  |

**Table S9 Clinical and molecular characteristics of TCGA HCC patients with a deep or shallow deletion of ALKBH5**

|  |  | **Deep or shallow deletion of ALKBH5** | | P |
| --- | --- | --- | --- | --- |
|  |  | yes | no |  |
| Age | <60 | 76 | 99 | 0.9800 |
|  | >60 | 84 | 110 |  |
| Gender | male | 108 | 144 | 0.8266 |
|  | female | 52 | 66 |  |
| Histological grade | G1 | 15 | 37 | **0.0330** |
|  | G2 | 76 | 101 |  |
|  | G3 | 58 | 65 |  |
|  | G4 | 9 | 4 |  |
| Pathological stage | I | 58 | 115 | **0.0033** |
|  | II | 45 | 42 |  |
|  | III | 41 | 40 |  |
|  | IV | 4 | 1 |  |
| T stage | T1 | 64 | 119 | **0.0062** |
|  | T2 | 51 | 44 |  |
|  | T3 | 36 | 41 |  |
|  | T4 | 8 | 4 |  |
|  | Tx | 1 | 0 |  |
| N stage | N0 | 107 | 147 | 0.0772 |
|  | N1 | 3 | 0 |  |
|  | Nx | 49 | 63 |  |
| M stage | M0 | 117 | 149 | 0.3260 |
|  | M1 | 3 | 1 |  |
|  | Mx | 40 | 60 |  |
| TP53 | alteration | 73 | 47 | **<0.0001** |
|  | wt | 86 | 159 |  |

**Table S10 Clinical and molecular characteristics of TCGA HCC patients with a deep or shallow deletion of FTO**

|  |  | **Deep or shallow deletion of FTO** | | P |
| --- | --- | --- | --- | --- |
|  |  | yes | no |  |
| Age | <60 | 86 | 89 | **0.0005** |
|  | >60 | 61 | 133 |  |
| Gender | male | 97 | 155 | 0.4771 |
|  | female | 50 | 68 |  |
| Histological grade | G1 | 13 | 39 | **0.0025** |
|  | G2 | 62 | 115 |  |
|  | G3 | 64 | 59 |  |
|  | G4 | 6 | 7 |  |
| Pathological stage | I | 59 | 114 | **0.0166** |
|  | II | 47 | 40 |  |
|  | III | 29 | 52 |  |
|  | IV | 2 | 3 |  |
| T stage | T1 | 64 | 119 | **0.0429** |
|  | T2 | 49 | 46 |  |
|  | T3 | 28 | 49 |  |
|  | T4 | 6 | 6 |  |
|  | Tx | 0 | 1 |  |
| N stage | N0 | 109 | 145 | >0.9999 |
|  | N1 | 1 | 2 |  |
|  | Nx | 36 | 76 |  |
| M stage | M0 | 115 | 151 | >0.9999 |
|  | M1 | 2 | 2 |  |
|  | Mx | 30 | 70 |  |
| TP53 | alteration | 60 | 60 | **0.0050** |
|  | wt | 85 | 160 |  |

**Table S11 Clinical and molecular characteristics of TCGA HCC patients with an amplification or copy number gain of YTHDF1**

|  |  | **Amplification or copy number gain of YTHDF1** | | P |
| --- | --- | --- | --- | --- |
|  |  | yes | no |  |
| Age | <60 | 54 | 121 | 0.2057 |
|  | >60 | 72 | 122 |  |
| Gender | male | 78 | 174 | 0.0658 |
|  | female | 48 | 70 |  |
| Histological grade | G1 | 8 | 44 | **0.0006** |
|  | G2 | 54 | 122 |  |
|  | G3 | 57 | 66 |  |
|  | G4 | 4 | 9 |  |
| Pathological stage | I | 51 | 122 | 0.1061 |
|  | II | 31 | 56 |  |
|  | III | 35 | 46 |  |
|  | IV | 3 | 2 |  |
| T stage | T1 | 52 | 131 | 0.0825 |
|  | T2 | 35 | 60 |  |
|  | T3 | 34 | 43 |  |
|  | T4 | 5 | 7 |  |
|  | Tx | 0 | 1 |  |
| N stage | N0 | 90 | 164 | >0.9999 |
|  | N1 | 1 | 2 |  |
|  | Nx | 35 | 77 |  |
| M stage | M0 | 97 | 169 | 0.1451 |
|  | M1 | 3 | 1 |  |
|  | Mx | 26 | 74 |  |
| TP53 | alteration | 43 | 77 | 0.5994 |
|  | wt | 81 | 164 |  |

**Table S12 Clinical and molecular characteristics of TCGA HCC patients with a deep or shallow deletion of YTHDF2**

|  |  | **Deep or shallow deletion of YTHDF2** | | P |
| --- | --- | --- | --- | --- |
|  |  | yes | no |  |
| Age | <60 | 69 | 106 | 0.5758 |
|  | >60 | 71 | 123 |  |
| Gender | male | 93 | 159 | 0.5886 |
|  | female | 47 | 71 |  |
| Histological grade | G1 | 14 | 38 | 0.1399 |
|  | G2 | 65 | 112 |  |
|  | G3 | 55 | 68 |  |
|  | G4 | 4 | 9 |  |
| Pathological stage | I | 58 | 115 | 0.3670 |
|  | II | 36 | 51 |  |
|  | III | 33 | 48 |  |
|  | IV | 3 | 2 |  |
| T stage | T1 | 63 | 120 | 0.5318 |
|  | T2 | 39 | 56 |  |
|  | T3 | 33 | 44 |  |
|  | T4 | 5 | 7 |  |
|  | Tx | 0 | 1 |  |
| N stage | N0 | 98 | 156 | 0.5620 |
|  | N1 | 2 | 1 |  |
|  | Nx | 40 | 72 |  |
| M stage | M0 | 108 | 158 | 0.3089 |
|  | M1 | 3 | 1 |  |
|  | Mx | 29 | 71 |  |
| TP53 | alteration | 34 | 86 | **0.0090** |
|  | wt | 104 | 141 |  |

**Table S13 Clinical and molecular characteristics of TCGA HCC patients with an amplification or copy number gain of YTHDF3**

|  |  | **Amplification or copy number gain of YTHDF3** | | P |
| --- | --- | --- | --- | --- |
|  |  | yes | no |  |
| Age | <60 | 99 | 76 | 0.0508 |
|  | >60 | 90 | 104 |  |
| Gender | male | 146 | 106 | **0.0002** |
|  | female | 44 | 74 |  |
| Histological grade | G1 | 17 | 35 | **0.0160** |
|  | G2 | 91 | 86 |  |
|  | G3 | 72 | 51 |  |
|  | G4 | 8 | 5 |  |
| Pathological stage | I | 89 | 84 | 0.3060 |
|  | II | 51 | 36 |  |
|  | III | 41 | 40 |  |
|  | IV | 1 | 4 |  |
| T stage | T1 | 91 | 92 | 0.4558 |
|  | T2 | 53 | 42 |  |
|  | T3 | 41 | 36 |  |
|  | T4 | 4 | 8 |  |
|  | Tx | 0 | 1 |  |
| N stage | N0 | 136 | 118 | 0.1030 |
|  | N1 | 0 | 3 |  |
|  | Nx | 54 | 58 |  |
| M stage | M0 | 141 | 125 | 0.3481 |
|  | M1 | 1 | 3 |  |
|  | Mx | 48 | 52 |  |
| TP53 | alteration | 63 | 57 | 0.7346 |
|  | wt | 124 | 121 |  |

**Table S14 Clinical and molecular characteristics of TCGA HCC patients with a shallow deletion of YTHDC1**

|  |  | **Shallow deletion of YTHDC1** | | P |
| --- | --- | --- | --- | --- |
|  |  | yes | no |  |
| Age | <60 | 84 | 91 | **0.0017** |
|  | >60 | 62 | 132 |  |
| Gender | male | 105 | 147 | 0.2043 |
|  | female | 41 | 77 |  |
| Histological grade | G1 | 15 | 37 | **0.0013** |
|  | G2 | 59 | 118 |  |
|  | G3 | 66 | 57 |  |
|  | G4 | 6 | 7 |  |
| Pathological stage | I | 68 | 105 | 0.5822 |
|  | II | 38 | 49 |  |
|  | III | 29 | 52 |  |
|  | IV | 3 | 2 |  |
| T stage | T1 | 71 | 112 | 0.7519 |
|  | T2 | 42 | 53 |  |
|  | T3 | 29 | 48 |  |
|  | T4 | 4 | 8 |  |
|  | Tx | 0 | 1 |  |
| N stage | N0 | 104 | 150 | >0.9999 |
|  | N1 | 1 | 2 |  |
|  | Nx | 40 | 72 |  |
| M stage | M0 | 115 | 151 | 0.3215 |
|  | M1 | 3 | 1 |  |
|  | Mx | 28 | 72 |  |
| TP53 | alteration | 78 | 42 | **<0.0001** |
|  | wt | 67 | 178 |  |

**Table S15 Clinical and molecular characteristics of TCGA HCC patients with an amplification or copy number gain of YTHDC2**

|  |  | **Amplification or copy number gain of YTHDC2** | | P |
| --- | --- | --- | --- | --- |
|  |  | yes | no |  |
| Age | <60 | 45 | 130 | **0.0318** |
|  | >60 | 70 | 124 |  |
| Gender | male | 88 | 164 | **0.0197** |
|  | female | 27 | 91 |  |
| Histological grade | G1 | 13 | 39 | 0.1612 |
|  | G2 | 59 | 118 |  |
|  | G3 | 34 | 89 |  |
|  | G4 | 7 | 6 |  |
| Pathological stage | I | 54 | 119 | 0.9174 |
|  | II | 28 | 59 |  |
|  | III | 23 | 58 |  |
|  | IV | 2 | 3 |  |
| T stage | T1 | 57 | 126 | 0.9596 |
|  | T2 | 31 | 64 |  |
|  | T3 | 24 | 53 |  |
|  | T4 | 3 | 9 |  |
|  | Tx | 0 | 1 |  |
| N stage | N0 | 75 | 179 | 0.5582 |
|  | N1 | 0 | 3 |  |
|  | Nx | 40 | 72 |  |
| M stage | M0 | 79 | 187 | 0.5858 |
|  | M1 | 2 | 2 |  |
|  | Mx | 34 | 66 |  |
| TP53 | alteration | 32 | 88 | 0.1562 |
|  | wt | 83 | 161 |  |

**Table S16 Clinical and molecular characteristics of TCGA HCC patients with an amplification or copy number gain of IGF2BP1**

|  |  | **Amplification or copy number gain of IGF2BP1** | | P |
| --- | --- | --- | --- | --- |
|  |  | yes | no |  |
| Age | <60 | 50 | 125 | 0.6987 |
|  | >60 | 59 | 135 |  |
| Gender | male | 81 | 171 | 0.0980 |
|  | female | 28 | 90 |  |
| Histological grade | G1 | 13 | 39 | 0.3113 |
|  | G2 | 47 | 130 |  |
|  | G3 | 44 | 79 |  |
|  | G4 | 4 | 9 |  |
| Pathological stage | I | 42 | 131 | 0.0856 |
|  | II | 24 | 63 |  |
|  | III | 32 | 49 |  |
|  | IV | 2 | 3 |  |
| T stage | T1 | 45 | 138 | **0.0498** |
|  | T2 | 28 | 67 |  |
|  | T3 | 30 | 47 |  |
|  | T4 | 6 | 6 |  |
|  | Tx | 0 | 1 |  |
| N stage | N0 | 75 | 179 | >0.9999 |
|  | N1 | 1 | 2 |  |
|  | Nx | 32 | 80 |  |
| M stage | M0 | 80 | 186 | 0.5873 |
|  | M1 | 2 | 2 |  |
|  | Mx | 27 | 73 |  |
| TP53 | alteration | 46 | 64 | **0.0018** |
|  | wt | 62 | 183 |  |

**Table S17 Clinical and molecular characteristics of TCGA HCC patients with an amplification or copy number gain of IGF2BP2**

|  |  | **Amplification or copy number gain of IGF2BP2** | | P |
| --- | --- | --- | --- | --- |
|  |  | yes | no |  |
| Age | <60 | 28 | 147 | 0.8976 |
|  | >60 | 32 | 162 |  |
| Gender | male | 45 | 207 | 0.2108 |
|  | female | 15 | 103 |  |
| Histological grade | G1 | 5 | 47 | **0.0310** |
|  | G2 | 24 | 153 |  |
|  | G3 | 25 | 98 |  |
|  | G4 | 5 | 8 |  |
| Pathological stage | I | 26 | 147 | 0.6022 |
|  | II | 17 | 70 |  |
|  | III | 13 | 68 |  |
|  | IV | 0 | 5 |  |
| T stage | T1 | 28 | 155 | 0.8849 |
|  | T2 | 18 | 77 |  |
|  | T3 | 12 | 65 |  |
|  | T4 | 2 | 10 |  |
|  | Tx | 0 | 1 |  |
| N stage | N0 | 37 | 217 | 0.3825 |
|  | N1 | 1 | 2 |  |
|  | Nx | 21 | 91 |  |
| M stage | M0 | 42 | 224 | >0.9999 |
|  | M1 | 0 | 4 |  |
|  | Mx | 18 | 82 |  |
| TP53 | alteration | 33 | 87 | **<0.0001** |
|  | wt | 26 | 219 |  |

**Table S18 Clinical and molecular characteristics of TCGA HCC patients with an amplification or copy number gain of IGF2BP3**

|  |  | **Amplification or copy number gain of IGF2BP3** | | P |
| --- | --- | --- | --- | --- |
|  |  | yes | no |  |
| Age | <60 | 46 | 129 | **0.0115** |
|  | >60 | 75 | 119 |  |
| Gender | male | 77 | 175 | 0.1982 |
|  | female | 44 | 74 |  |
| Histological grade | G1 | 13 | 39 | 0.6265 |
|  | G2 | 59 | 118 |  |
|  | G3 | 42 | 81 |  |
|  | G4 | 5 | 8 |  |
| Pathological stage | I | 55 | 118 | 0.7722 |
|  | II | 28 | 59 |  |
|  | III | 30 | 51 |  |
|  | IV | 1 | 4 |  |
| T stage | T1 | 58 | 125 | 0.8574 |
|  | T2 | 33 | 62 |  |
|  | T3 | 27 | 50 |  |
|  | T4 | 3 | 9 |  |
|  | Tx | 0 | 1 |  |
| N stage | N0 | 79 | 175 | 0.2345 |
|  | N1 | 2 | 1 |  |
|  | Nx | 40 | 72 |  |
| M stage | M0 | 92 | 174 | >0.9999 |
|  | M1 | 1 | 3 |  |
|  | Mx | 28 | 72 |  |
| TP53 | alteration | 42 | 78 | 0.4941 |
|  | wt | 77 | 168 |  |

**Table S19 Clinical and molecular characteristics of TCGA HCC patients with an amplification or copy number gain of HNRNPA2B1**

|  |  | **Amplification or copy number gain of HNRNPA2B1** | | P |
| --- | --- | --- | --- | --- |
|  |  | yes | no |  |
| Age | <60 | 45 | 130 | **0.0188** |
|  | >60 | 72 | 122 |  |
| Gender | male | 73 | 179 | 0.1087 |
|  | female | 44 | 74 |  |
| Histological grade | G1 | 12 | 40 | 0.4932 |
|  | G2 | 56 | 121 |  |
|  | G3 | 42 | 81 |  |
|  | G4 | 5 | 8 |  |
| Pathological stage | I | 52 | 121 | 0.6569 |
|  | II | 27 | 60 |  |
|  | III | 30 | 51 |  |
|  | IV | 1 | 4 |  |
| T stage | T1 | 55 | 128 | 0.7905 |
|  | T2 | 32 | 63 |  |
|  | T3 | 27 | 50 |  |
|  | T4 | 3 | 9 |  |
|  | Tx | 0 | 1 |  |
| N stage | N0 | 79 | 175 | 0.2345 |
|  | N1 | 2 | 1 |  |
|  | Nx | 36 | 76 |  |
| M stage | M0 | 89 | 177 | >0.9999 |
|  | M1 | 1 | 3 |  |
|  | Mx | 27 | 73 |  |
| TP53 | alteration | 39 | 81 | 0.7750 |
|  | wt | 76 | 169 |  |

**Table S20 Clinical and molecular characteristics of TCGA HCC patients with an amplification or copy number gain of HNRNPC**

|  |  | **Shallow deletion of HNRNPC** | | P |
| --- | --- | --- | --- | --- |
|  |  | yes | no |  |
| Age | <60 | 44 | 131 | 0.0968 |
|  | >60 | 35 | 159 |  |
| Gender | male | 58 | 194 | 0.2535 |
|  | female | 21 | 97 |  |
| Histological grade | G1 | 5 | 47 | 0.0542 |
|  | G2 | 37 | 140 |  |
|  | G3 | 31 | 92 |  |
|  | G4 | 5 | 8 |  |
| Pathological stage | I | 27 | 146 | **0.0164** |
|  | II | 22 | 65 |  |
|  | III | 22 | 59 |  |
|  | IV | 3 | 2 |  |
| T stage | T1 | 28 | 155 | **0.0373** |
|  | T2 | 26 | 69 |  |
|  | T3 | 22 | 55 |  |
|  | T4 | 3 | 9 |  |
|  | Tx | 0 | 1 |  |
| N stage | N0 | 56 | 198 | 0.5303 |
|  | N1 | 1 | 2 |  |
|  | Nx | 22 | 90 |  |
| M stage | M0 | 64 | 202 | 0.2515 |
|  | M1 | 2 | 2 |  |
|  | Mx | 13 | 87 |  |
| TP53 | alteration | 43 | 77 | **<0.0001** |
|  | wt | 35 | 210 |  |

**Table S21 Correlation between the METTL16 expression and clinicopathologic features of HCC patients (n=66)**

|  |  | **METTL16 expression** | | P-value |
| --- | --- | --- | --- | --- |
|  |  | High (n=33) | Low (n=33) |  |
| Age | <60 | 20 | 23 | 0.6059 |
|  | >60 | 13 | 10 |  |
| Gender | male | 30 | 27 | 0.4752 |
|  | female | 3 | 6 |  |
| Pathological grade | I-II | 22 | 24 | 0.5922 |
|  | III | 11 | 9 |  |
| Clinial stage | 1/2 | 31 | 26 | 0.1487 |
|  | 3/4 | 2 | 7 |  |
| Tumor size | <5cm | 24 | 20 | 0.4399 |
|  | >5cm | 9 | 13 |  |
