# Supplementary material for: Gene Signatures and Prognostic Values of m6A Regulators in Hepatocellular Carcinoma
Source: Front Genet. 2020 Oct 2;11:540186. doi: 10.3389/fgene.2020.540186 (PMC7567013; doi:10.3389/fgene.2020.540186)
Supplement: Supplementary file 2 [file Data_Sheet_2.docx]

**Supplementary Figure legends**

**Figure S1** Kaplan-Meier curves for overall survival of HCC patients with different CNV patterns of m6A regulators METTL3, METTL14, WTAP, KIAA1429, RBM15, ZC3H13, FTO and HNRNPC.

**Figure S2** Kaplan-Meier curves for overall survival of HCC patients with different CNV patterns of m6A regulators YTHDF1, YTHDF2, YTHDF3, YTHDC1, YTHDC2, IGF2BP1, IGF2BP2, IGF2BP3 and HNRNPA2B1.

**Figure S3** IHC staining of METTL16 in normal livers (A-C) and HCC (D-I) tissues in the HPA database.

**Figure S4** The copy number of METTL16 gene was decreased in HCC than that in normal livers. The data was obtained from Oncomine database (Guichard’s HCC cohort).

**Figure S5** Kaplan-Meier curve for overall survival of TCGA HCC patients according to the high or low level of METTL16 mRNA. The graph was generated by Kaplan-Meier plotter (http://www.kmplot.com/analysis/index.php?p=service&cancer=liver_rnaseq).

**Figure S6** Expression of genes related to peroxisome and PPAR signaling pathway in HCC. Data was obtained from the Gene Expression Profiling Interactive Analysis (GEPIA) database (<http://gepia.caner-pku.cn/>). “*”p<0.05.
