# Supplementary figures and images for: Gene Signatures and Prognostic Values of m6A Regulators in Hepatocellular Carcinoma
Source: Front Genet. 2020 Oct 2;11:540186. doi: 10.3389/fgene.2020.540186 (PMC7567013; doi:10.3389/fgene.2020.540186)

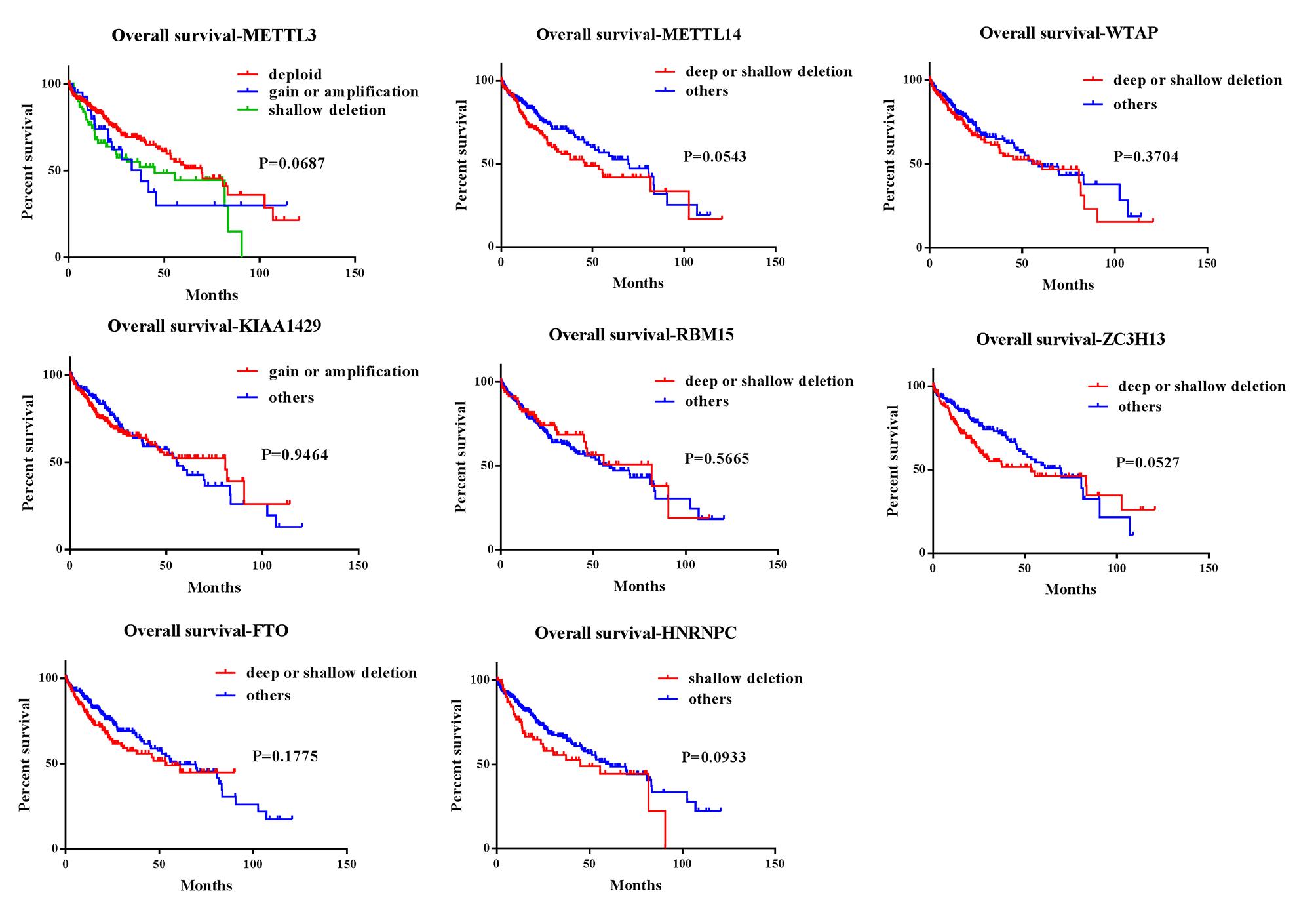

Supplement: Supplementary file 3 [file Image_1.TIF]

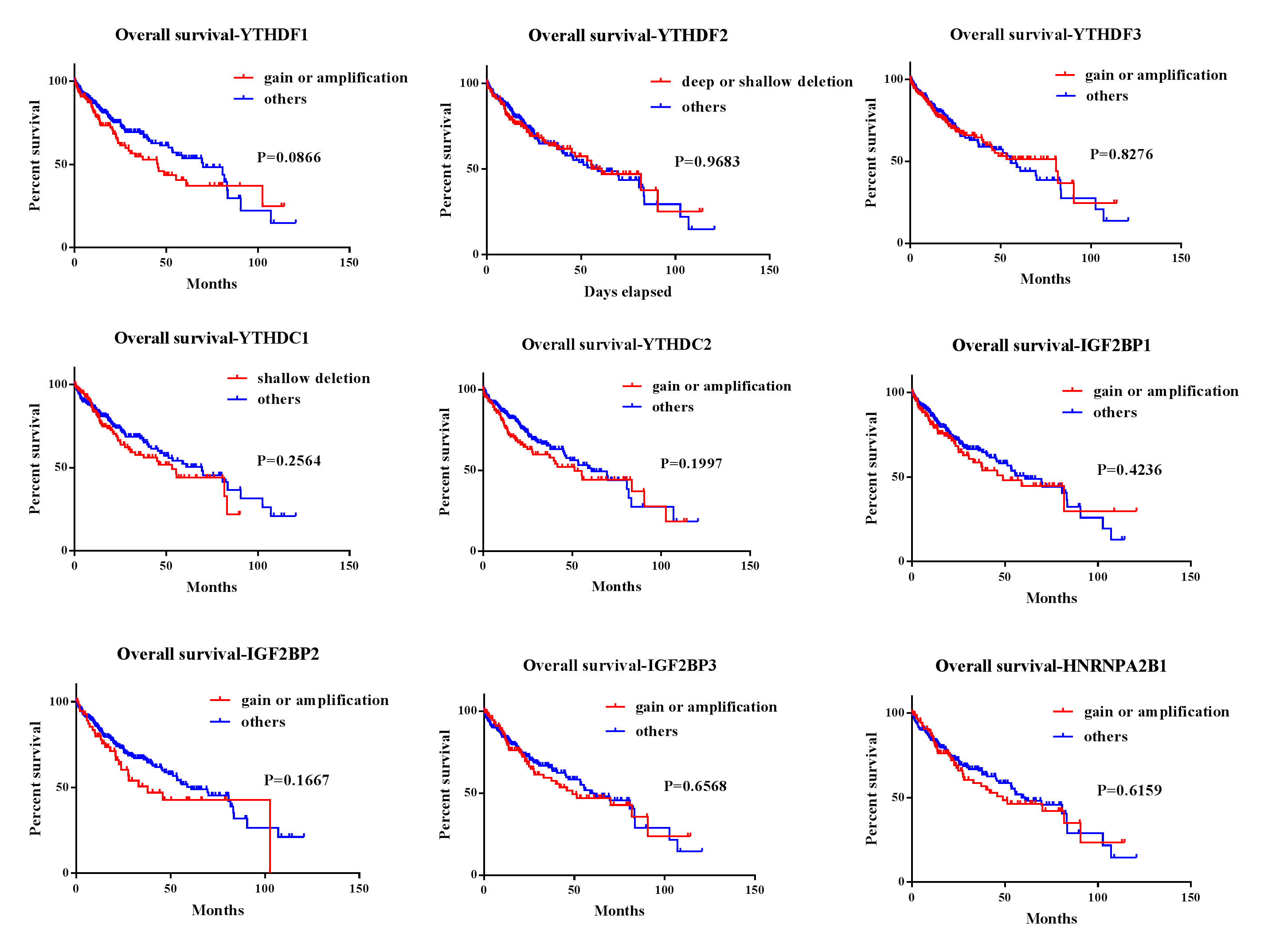

Supplement: Supplementary file 4 [file Image_2.TIF]

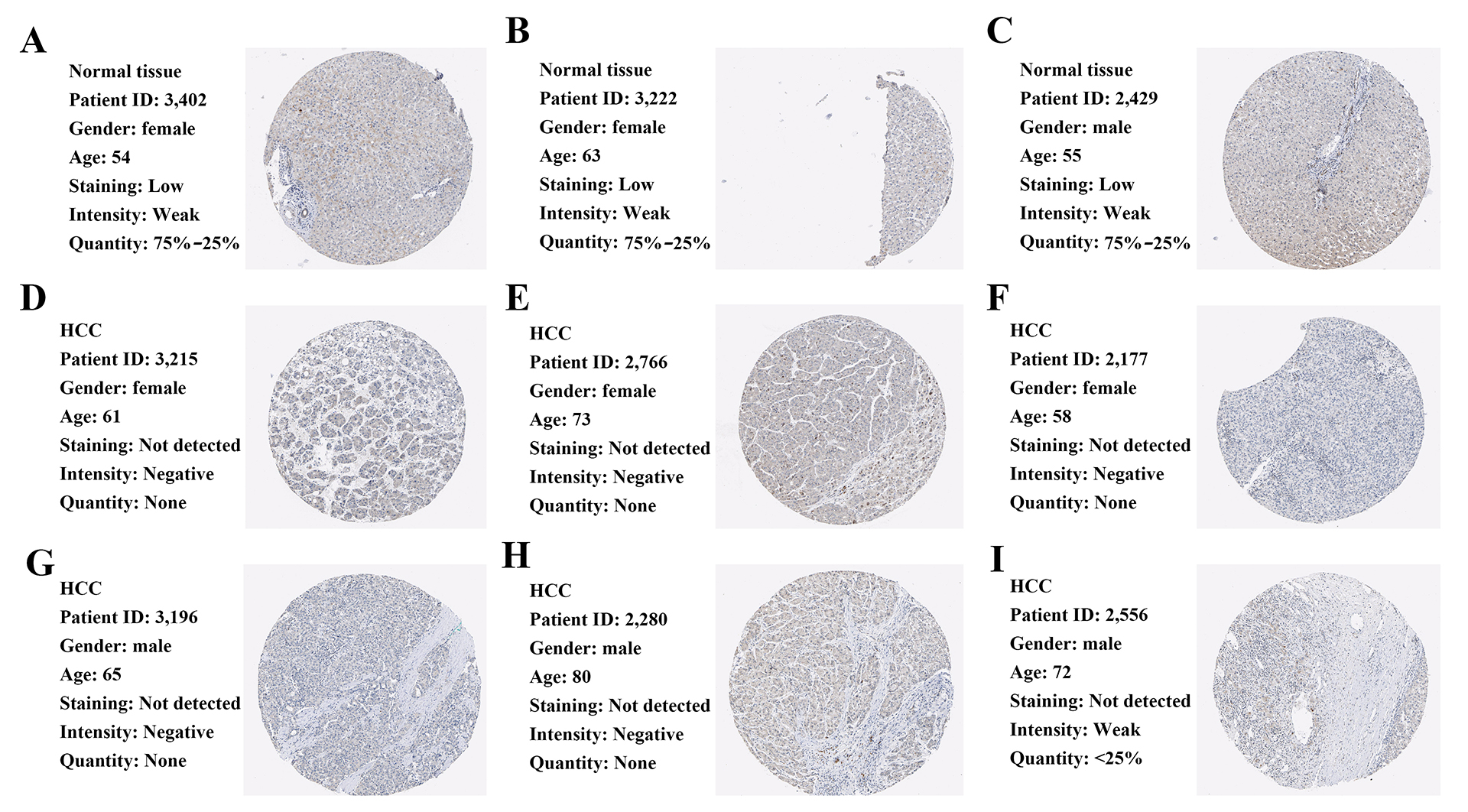

Supplement: Supplementary file 5 [file Image_3.JPEG]

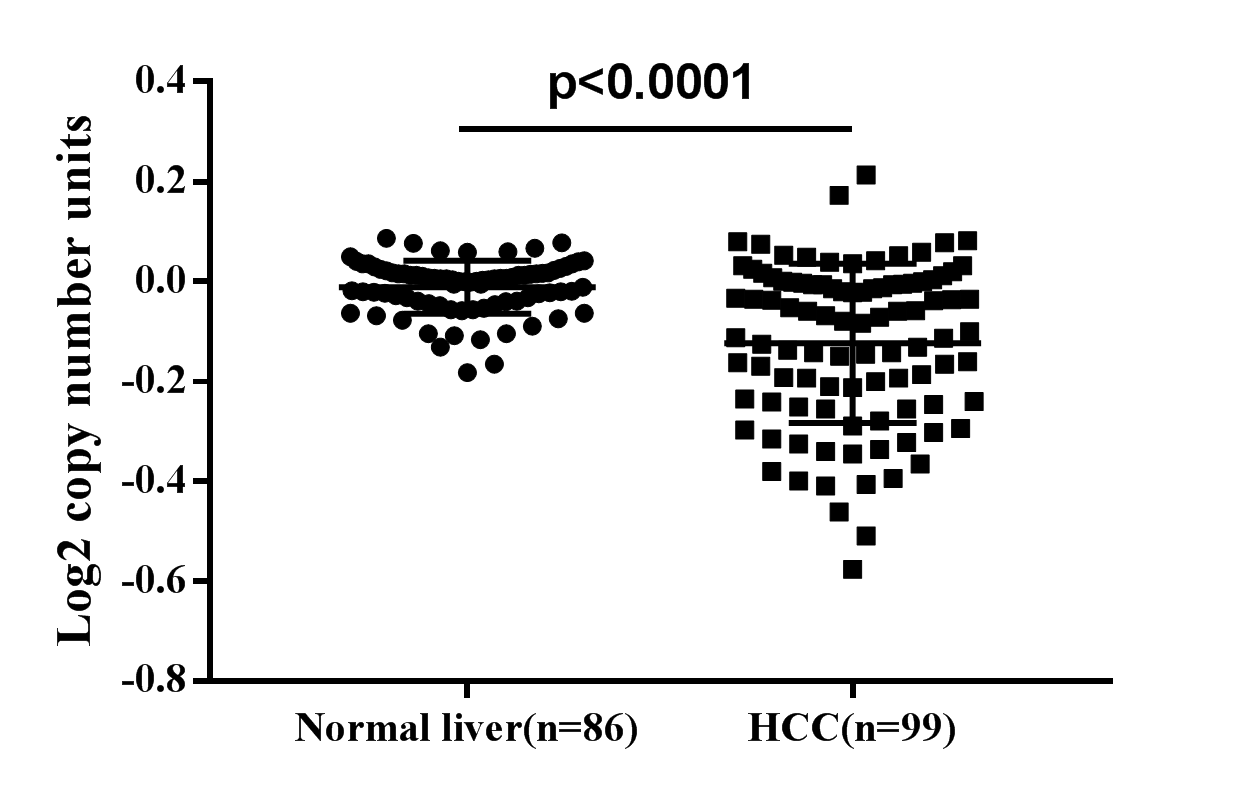

Supplement: Supplementary file 6 [file Image_4.TIF]

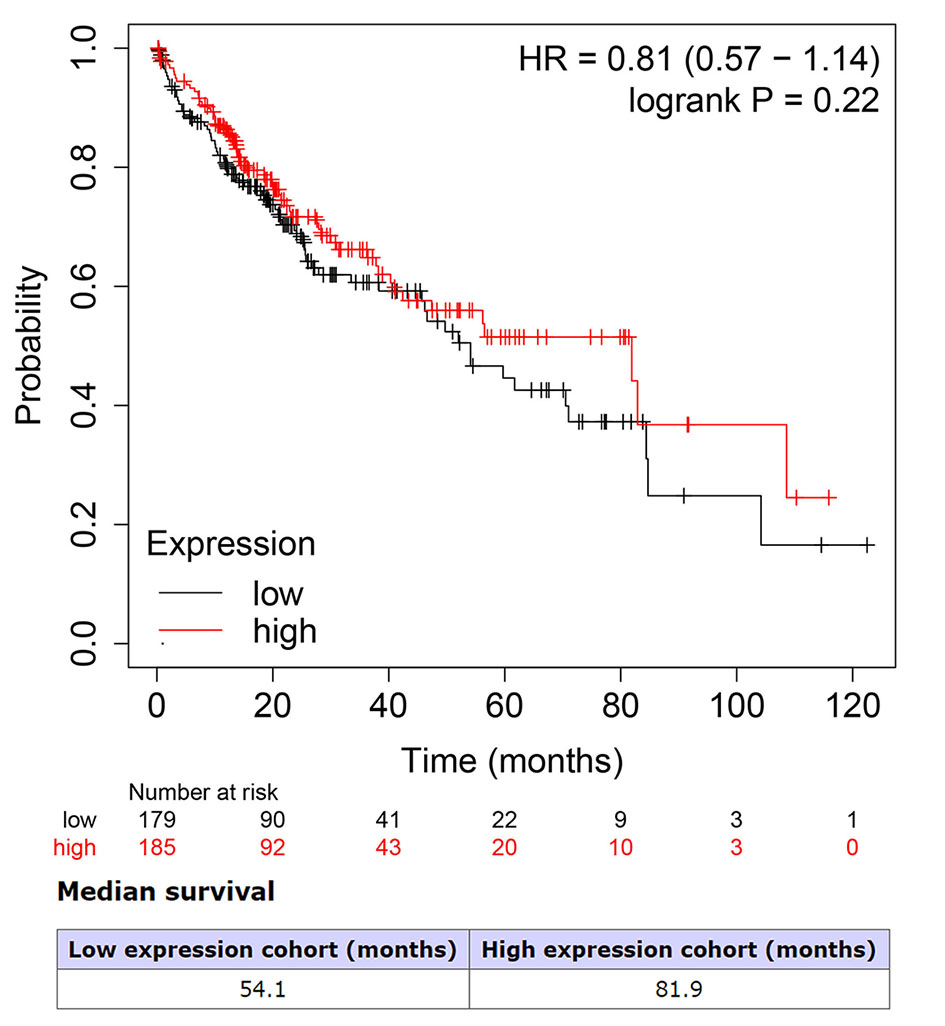

Supplement: Supplementary file 7 [file Image_5.JPEG]

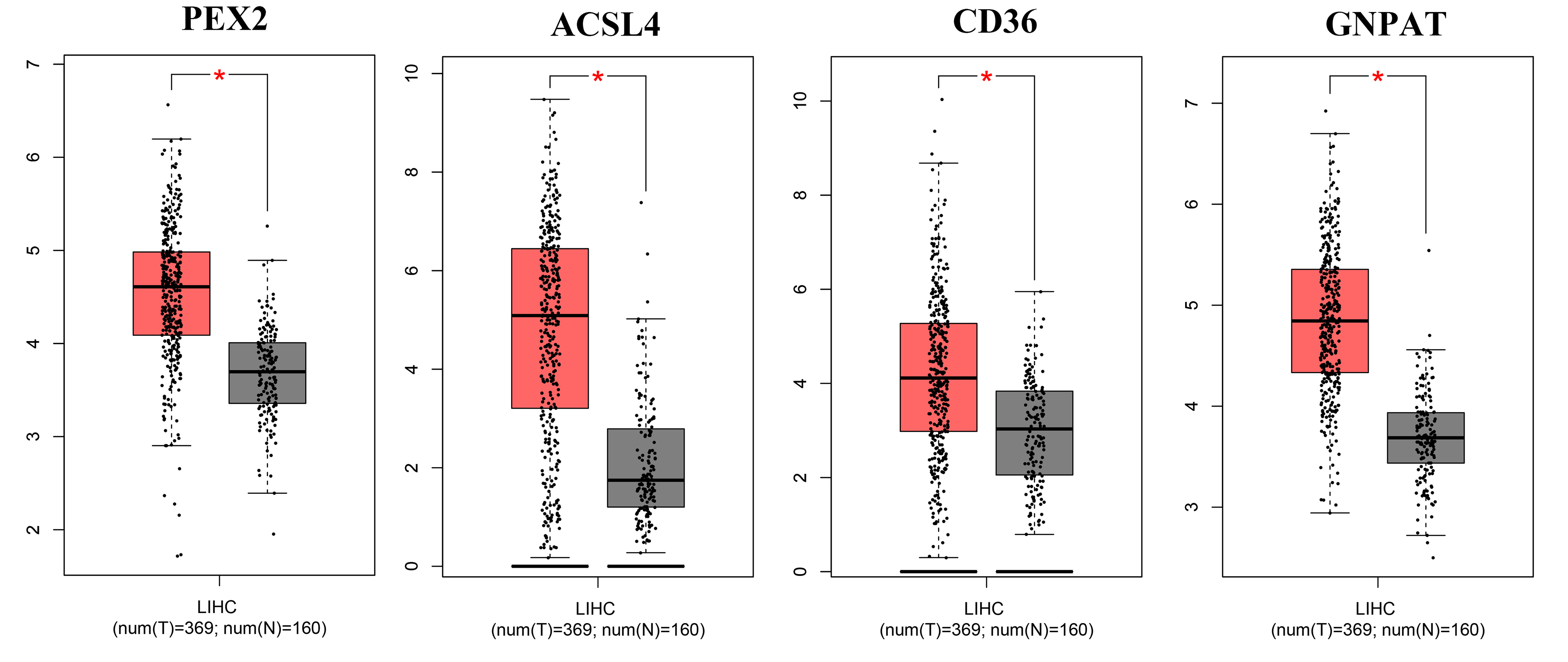

Supplement: Supplementary file 8 [file Image_6.JPEG]
